# Improving access to antidotes and antivenoms, Thailand

**DOI:** 10.2471/BLT.18.217075

**Published:** 2018-10-29

**Authors:** Netnapis Suchonwanich, Winai Wananukul

**Affiliations:** a National Health Security Office, Bangkok, Thailand.; b Faculty of Medicine, Ramathibodi Hospital, 270 Rama 6 Road, Thung Phayathai Subdistrict, Ratchathewi District, Bangkok, 10400, Thailand.

## Abstract

**Problem:**

Historically in Thailand, access to poison antidotes was limited and antivenom stock management was inefficient.

**Approach:**

In 2010, the country established a national antidote programme, which created national and subnational antidote stocks, managed their distribution and trained health-care providers on clinical management and antidote use. In 2013, the programme incorporated antivenoms to improve stock management and avoid wastage due to stock expiry.

**Local setting:**

Before the programme, health-care providers consulted poison centres on clinical management of poisoning and some antidotes were not available. Individual hospitals stocked antivenoms, which often expired before use.

**Relevant changes:**

Today, the National Health Security Office finances and manages the centralized procurement of antidotes and antivenoms and all Thai patients have a right to antidotes regardless of health insurance. National and subnational stock levels are determined based on demand, treatment urgency and cost. A web-based system, which incorporates geographical information, was introduced for requesting antidotes and antivenoms. Poison centres provide training, 24-hour consultation services and outcome monitoring. Antidotes and antivenoms are now readily available and used correctly and clinical management has improved. Moreover, better stock and distribution control has helped avoid antivenom wastage and reduced antivenom costs, from US$ 2.23 million United States dollars (US$) to US$ 1.2 million.

**Lessons learnt:**

The programme’s success depended on strong and sustained policy support, adequate funding, improved operational capacity, training for health-care professionals and the provision of 24-hour online consultation services. A web-based centralized procurement and distribution ensured these essential medicines were available, minimized costs, reduced waste and saved lives.

## Résumé

**Problème:**

Historiquement, en Thaïlande, l'accès aux antidotes était limité et la gestion des stocks d'antivenins peu efficace.

**Approche:**

En 2010, le pays a mis en place un programme national relatif aux antidotes, qui a permis de créer des stocks nationaux et infranationaux d'antidotes, de gérer leur distribution et de former les prestataires de soins à l'usage des antidotes et à la prise en charge clinique des patients. En 2013, le programme a intégré les antivenins afin d'améliorer la gestion des stocks et d'éviter le gaspillage dû à leur expiration.

**Environnement local:**

Avant la mise en place du programme, les prestataires de soins consultaient les centres antipoison au sujet de la prise en charge clinique des intoxications et certains antidotes n'étaient pas disponibles. Les hôpitaux stockaient des antivenins, qui expiraient souvent avant d'être utilisés.

**Changements significatifs:**

Aujourd'hui, le Bureau national de sécurité sanitaire finance et gère l'approvisionnement centralisé en antidotes et en antivenins et tous les patients thaïlandais peuvent bénéficier d'antidotes, quelle que soit leur assurance maladie. Les quantités des stocks nationaux et infranationaux sont déterminées en fonction de la demande, de l'urgence des traitements et des coûts. Un système en ligne intégrant des informations géographiques a été mis en place pour demander des antidotes et des antivenins. Les centres antipoison assurent des formations, des consultations 24 h/24 ainsi qu'un suivi des patients. Des antidotes et des antivenins sont désormais rapidement disponibles et correctement utilisés, et la prise en charge clinique s'est améliorée. Par ailleurs, le meilleur contrôle des stocks et de la distribution a permis d'éviter le gaspillage des antivenins et de réduire les coûts associés, qui sont passés de 2,23 millions de dollars des États-Unis ($ US) à 1,2 million de $ US.

**Leçons tirées:**

La réussite du programme a découlé du soutien politique fort et constant, du financement adéquat, de l'amélioration des capacités opérationnelles, de la formation des professionnels de santé et de la prestation de services de consultation en ligne 24 heures sur 24. Un système en ligne d'approvisionnement et de distribution centralisés a permis d'assurer la disponibilité de ces médicaments essentiels, de réduire les coûts ainsi que le gaspillage et de sauver des vies.

## Mejorar el acceso a los antídotos y antisueros, Tailandia

Resumen

**Problema:**

Históricamente en Tailandia, el acceso a los antídotos venenosos era limitado y la gestión de las existencias de antisueros era ineficaz.

**Enfoque:**

En 2010, el país estableció un programa nacional de antídotos, que creó reservas nacionales y subnacionales de antídotos, gestionó su distribución y formó a los profesionales sanitarios en la gestión clínica y el uso de antídotos. En 2013, el programa incorporó antisueros para mejorar la gestión de las existencias y evitar el despilfarro debido a la expiración de las existencias.

**Entorno local:**

Antes del programa, los profesionales sanitarios consultaron a los centros de intoxicación sobre el tratamiento clínico de la intoxicación y algunos antídotos no estaban disponibles. Los hospitales individuales almacenaban antisueros, que a menudo expiraban antes de su uso.

**Cambios relevantes:**

En la actualidad, la Oficina Nacional de Seguridad Sanitaria financia y gestiona la adquisición centralizada de antídotos y antisueros, y todos los pacientes tailandeses tienen derecho a los antídotos, independientemente del seguro médico. Los niveles de existencias nacionales y subnacionales se determinan en función de la demanda, la urgencia del tratamiento y el coste. Se introdujo un sistema basado en la web, que incorpora información geográfica, para solicitar antídotos y antisueros. Los centros de intoxicación ofrecen formación, servicios de consulta las 24 horas y seguimiento de los resultados. Los antídotos y los antisueros están ahora disponibles y se utilizan correctamente y la gestión clínica ha mejorado. Además, un mejor control de las existencias y la distribución ha contribuido a evitar el despilfarro de antisueros y a reducir los costes de los mismos, de 2,23 millones de dólares a 1,2 millones de dólares estadounidenses.

**Lecciones aprendidas:**

El éxito del programa dependía de un apoyo político sólido y sostenido, de una financiación adecuada, de una mayor capacidad operativa, de la formación de los profesionales sanitarios y de la prestación de servicios de consulta en línea las 24 horas del día. La adquisición y distribución centralizadas a través de Internet garantizaron la disponibilidad de estos medicamentos esenciales, minimizaron los costes, redujeron los desechos y salvaron vidas.

## ملخص

**المشكلة:**

كان الوصول في تايلند إلى ترياقات السموم محدودًا على مدى التاريخ، وكانت إدارة المخزون من مضادات السموم غير فعالة.

**الأسلوب:**

في عام 2010، أنشأت الدولة برنامجًا وطنيًا للترياقات، وهو ما أدى إلى إنشاء مخزونات وطنية ودون وطنية من الترياقات، وتولى إدارة توزيعها وتدريب مقدمي الرعاية الصحية على الإدارة السريرية واستخدام الترياق. في عام 2013، أدرج البرنامج مضادات السموم لتحسين إدارة المخزون وتجنب الفاقد بسبب انتهاء صلاحية المخزون.

**المواقع المحلية:**

قبل البرنامج، قام مقدمو الرعاية الصحية باستشارة مراكز السموم بشأن الإدارة السريرية لتوريد العلاجات، ولم تكن بعض الترياقات متاحة. قامت المستشفيات الفردية بتخزين مضادات السموم، والتي غالبًا ما تنتهي صلاحيتها قبل الاستخدام.

**التغيّرات ذات الصلة:**

اليوم، يقوم المكتب الوطني للأمن الصحي بتمويل وإدارة المشتريات المركزية للترياقات ومضادات السموم، ولجميع المرضى التايلنديين الحق في الحصول على الترياقات بغض النظر عن التأمين الصحي. يتم تحديد مستويات المخزون الوطني ودون الوطني بناء على الطلب وإلحاح المعالجة والتكلفة. تم طرح نظام على شبكة الإنترنت، يتضمن معلومات جغرافية، لطلب الترياقلت ومضادات السموم. توفر مراكز السموم التدريب، وخدمات الاستشارات على مدار 24 ساعة يومياً، فضلاً عن مراقبة النتائج. أصبحت الترياقات ومضادات السموم متوفرة الآن بشكل متيسر، وتستخدم بشكل صحيح، كما تحسنت الإدارة السريرية. علاوة على ذلك، ساعدت المراقبة الأفضل للمخزون والتوزيع على تجنب إهدار مضادات السموم وتخفيض تكاليفها، من 2.23 مليون دولار أمريكي إلى 1.2 مليون دولار أمريكي.

**الدروس المستفادة:**

اعتمد نجاح البرنامج على الدعم القوي والمستدام للسياسات، والتمويل الكافي، وتحسين القدرة التشغيلية، وتدريب أخصائيي الرعاية الصحية، وتوفير خدمات الاستشارات عبر الإنترنت على مدار 24 ساعة يومياً. وعن طريق الشراء والتوزيع المركزيين اللذين يعتمدان على شبكة الإنترنت، تم ضمان توفر هذه الأدوية الأساسية وتقليص التكاليف وتقليل الإهدار وحفظ الأرواح.

## 摘要

**问题:**

自古至今，在泰国，获取解毒剂的方法有限，且抗蛇毒血清的库存管理效率低下。

**方法:**

2010 年，该国建立了国家解毒剂项目，创建了国家和地方的解毒剂库存中心，管理其分配，并对临床管理和解毒剂使用方面的医疗护理提供人员进行培训。2013 年，该项目纳入抗蛇毒血清，以改善库存管理，避免因库存过期而造成的浪费。

**当地状况:**

在该项目实施之前，医疗护理提供人员就中毒的临床处理咨询了中毒控制中心，但某些解毒剂在当时无法提供。个别医院备有抗蛇毒血清，但通常尚未使用就已经过期。

**相关变化:**

如今，国家健康安全局 (National Health Security Office) 对解毒剂和抗蛇毒血清的集中采购进行资助和管理，所有的泰国患者都有权在不考虑医疗保险的情况下获取解毒剂。国家和地方的库存水平是根据需求、治疗的紧迫性和成本确定的。它是一个基于网络的系统，纳入了地理信息，是为了索取解毒剂和抗蛇毒血清而引进的。中毒控制中心提供培训、24 小时咨询服务和结果监测。如今，解毒剂和抗蛇毒血清易于获取、得以正确使用，临床管理也得到了改善。此外，更好的库存和分配控制有助于避免抗蛇毒血清的浪费，将抗蛇毒血清的成本从 223 万美元 降至 120 万美元。

**经验教训:**

该项目的成功取决于强有力和持续的政策支持、充足的资金、日益改善的操作水平，对医疗护理专业人员的培训和 24 小时在线咨询服务的提供。基于网络的集中采购和分配确保了此类基本药物的供应，最大化地降低了成本，从而减少浪费、挽救生命。****

## Резюме

**Проблема:**

Исторически доступ к противоядиям в Таиланде был ограничен и управление запасами противозмеиных сывороток осуществлялось неэффективно.

**Подход:**

В 2010 году в стране была развернута национальная программа по противоядиям, благодаря которой были созданы национальные и субнациональные запасы противоядий, налажено управление их распространением и обучение работников системы здравоохранения клиническому ведению случаев отравления и использованию противоядий. В 2013 году в программу были включены противозмеиные сыворотки с целью совершенствования управления запасами и во избежание потерь по причине истечения срока годности запасов.

**Местные условия:**

До начала программы работники здравоохранения консультировали центры по борьбе с отравлениями относительно лечения пострадавших, при этом некоторые противоядия были недоступны. В отдельных больницах были запасы противозмеиной сыворотки, которые часто не использовались и имели истекший срок годности.

**Осуществленные перемены:**

Сегодня Национальная служба безопасности здоровья финансирует централизованные закупки противоядий и противозмеиных сывороток и управляет этим процессом и все пациенты в Таиланде имеют право на получение противоядия, независимо от наличия у них страховки. Национальные и субнациональные уровни запасов определяются на основании потребности, срочности лечения и затрат. На основе географических данных была внедрена сетевая система для подачи заявок на получение противоядий и противозмеиных сывороток. Центры по борьбе с отравлениями обеспечивают обучение, круглосуточные консультации и мониторинг исходов. Противоядия и противозмеиные сыворотки теперь всегда доступны, используются правильно, а также улучшился клинический уход за пострадавшими. Более того, улучшение контроля запасов и распределения противоядий помогло предотвратить порчу противозмеиных сывороток и снизило расходы на этот вид препаратов с 2,23 до 1,2 миллиона долларов США.

**Выводы:**

Успех программы зависел от сильной и неизменной поддержки стратегии, достаточного финансирования, улучшенного оперативного потенциала, обучения медработников и предоставления круглосуточных услуг консультантов в сети. Централизованный режим закупок и распределения, основанный на использовании сети, гарантировал доступность этих жизненно важных лекарственных препаратов, позволил минимизировать затраты, снизить порчу лекарств и спасти много жизней.****

## Introduction

According to the Ramathibodi Poison Center in Thailand, there are more than 15 000 cases of poisoning in the country each year.^1^ However, the true figure may be higher because consultations with poison centres are optional. Antidotes are important for treatment and, when they are unavailable, treatment efficacy may be reduced and outcomes compromised.^2^ Unfortunately, many antidotes are not readily available. In fact, they have been referred to as orphan drugs due to their scarcity.^3^^,^^4^

In 2002, Thailand achieved universal health coverage when the whole population gained access to three public health insurance systems.^5^ The National Health Security Office was established by law to manage the Universal Health Coverage Scheme,^5^ which caters for 75% of the population and receives an annual budget funded through general taxation. Although the scheme is comprehensive and includes high-cost medicines,^6^ ensuring access to antidotes was initially a challenge since supplies were limited and there was no active management system. In contrast, antivenoms were readily available throughout the country because all hospitals had stocks. However, management was inefficient and some antivenoms passed their expiry dates before they could be used.

The need to improve access to antivenoms has been recognized as a major challenge for low- and middle-income countries and in 2018, the World Health Assembly adopted a resolution on the burden of snakebite envenoming.^7^ Here we describe the lessons learnt in Thailand with a programme designed to increase access to both antidotes and antivenoms and to improve the efficiency of antivenom stock management.

## Local setting

The market for antidotes is small, often their patents have expired, demand is unpredictable and profit margins are low. Consequently, the pharmaceutical industry has little incentive to develop and market these medicines, even though they are used for life-threatening conditions. Moreover, antidotes are expensive and have a short shelf life, which discourages individual hospitals from keeping large stocks. The World Health Organization (WHO) recommends establishing a central bank of antidotes as an effective and efficient way of ensuring prompt access to these drugs, thereby saving lives.^8^

In 2002, the National Health Security Office in Thailand set up a special system to improve access to high-cost medicines.^6^ Medicines were selected and procured centrally, and management of the supply chain was outsourced by the Government Pharmaceutical Organization to the private sector. This resulted in annual savings of a few billion Thai baht (i.e. 50 to 100 million United States dollars; US$).^9^^,^^10^ Although this system improved access to high-cost medicines from 2009 onwards,^6^ access to antidotes remained a major challenge. In contrast, access to antivenoms was not a problem, but there was considerable wastage because all hospitals held stocks and drugs often expired before use.

## National antidote programme

In 2010, the National Health Security Board established a national antidote programme to ensure equitable access to antidotes for the whole population, not only members of the Universal Health Coverage Scheme. The programme made extensive use of information and communications technology, not only to support procurement and supply chain management, but also for teleconsultations, which enabled frontline health professionals, particularly those in remote areas, to get advice on the proper use of antidotes and on clinical management.

Under the programme, national and subnational stocks of antidotes were established as hubs for supplies. The hubs content is based on three criteria: (i) demand in the local area; (ii) whether an antidote had to be provided urgently; and (iii) cost. Today hospitals request antidotes through a web-based system with a high level of data integrity and security. This system includes data on real-time inventories at all hubs and on expiry dates. The distribution of antidotes to hospitals that request them is managed by the stocking centres and responsibility for replenishing stocks has been outsourced by the Government Pharmaceutical Organization to the private sector. The programme is also involved in training health-care providers on the appropriate use of antidotes: manuals and clinical guidelines have been circulated and in-service training is organized annually (Box 1).

Box 1 **Components of the Thai national antidote programme, 2010**

**Financing**

- Continued political support ensured that the National Health Security Office received full funding for antidotes and antivenoms for the whole population, thereby protecting against financial risks and saving lives.

**Design and operations**

- Antidotes and antivenoms were stocked nationally and subnationally, as guided by epidemiological evidence.
- Procurement and supply chain management became more efficient: antidotes and antivenoms were provided rapidly following web-based requests and delivered through vendor-managed inventory systems.
- Efficient procurement and management of antivenoms resulted in substantial cost savings compared with the previous system in which individual hospitals purchased and stocked antivenoms, with the danger of stock expiring.

**Capacity building**

- Staff capacity was increased through: (i) the circulation of manuals and guidelines; (ii) annual in-service training for health-care professionals; (iii) the establishment of a 24-hour, online, real-time, clinical consultation service to support case management remotely; and (iv) outcome monitoring.

The programme involves close collaboration between several agencies: (i) the National Health Security Office allocates the annual budget and steers and monitors the programme; (ii) the Queen Saovabha Memorial Institute produces antivenom and some antidotes; (iii) the Government Pharmaceutical Organization is responsible for procurement and for managing the supply chain; (iv) the Thai Food and Drug Administration registers medical products; (v) the Thai Society of Clinical Toxicology provides clinical expertise and training; (vi) poison centres at Siriraj and Ramathibodi Hospitals provide clinical consultations; and (vii) the Ramathibodi Poison Center is responsible for monitoring treatment outcomes.

Initially, six antidotes were selected using epidemiological data from the Ramathibodi Poison Center: sodium nitrite, sodium thiosulfate, methylene blue, dimercaprol, succimer and glucagon. Succimer, dimercaprol and glucagon were procured from abroad by the Government Pharmaceutical Organization. Methylene blue, sodium nitrite and sodium thiosulfate were mainly manufactured by the Queen Saovabha Memorial Institute at a price per vial 30 times lower than imported equivalents.

Antidotes were divided into four categories: (i) critical antidotes, which must be administered in less than 1 hour; (ii) emergency antidotes, to be administered within 1 to 6 hours; (iii) urgent antidotes, to be administered within 6 to 24 hours; and (iv) non-urgent antidotes. In 2018, botulinum antitoxin was held at the Ramathibodi Poison Center only as demand was small and the antidote is very expensive (i.e. up to US$ 12 500 per treatment course). Whereas cyanide antidotes were held in subnational stocks since demand was homogenous throughout the country and dimercaprol was held at only a few subnational centres because demand was low and patients were treated in well-equipped hospitals.

Before the programme, individual hospitals procured and stocked snake antivenom themselves without adequate data on poisonous snakes in their localities. Consequently, supply exceeded demand and antivenom was wasted. In response, antivenom was integrated into the programme in 2013 and subnational stocks were adjusted in accordance with local epidemiological data on snake bite cases. This improved the efficiency of stock management and minimized waste. The Queen Saovabha Memorial Institute plays a principal role in producing antivenoms.

General and regional hospitals were invited to serve as subnational centres for stocks of antidotes and antivenoms. In addition, these drugs were included in the national list of essential medicines, which means they are covered by all three public health insurance schemes and their cost is incorporated into annual budgets. As clinical management is as important as the availability of antidotes, annual training for health-care professionals on the use of antidotes was initiated and guidelines on antidote use were distributed. Importantly, poison centres were made responsible for supervising treatment with antidotes and for monitoring clinical outcomes. The operation of the programme is continually being improved with the help of evaluations and reviews. Currently, the programme covers nine antidotes and seven antivenoms.

## Relevant changes

Since the programme was implemented in 2011, there has been no shortage of any antidote or antivenom covered. Previously, almost no antidotes were available and deaths occurred. Between 2011 and 2017, 1800 patients who were poisoned benefited from the programme. In addition, 25 636 patients exposed to snake venom had access to the appropriate antivenom and lives were saved. Adjusting the subnational stocking and distribution of antivenom to match the local prevalence of snakebite cases resulted in more efficient stock management and reduced costs: the average annual procurement budget for antivenom decreased from US$ 2.23 million in 2012 when all hospitals purchased their own antivenom to US$ 1.2 million between 2013 and 2017, a 46% cost saving despite relatively constant demand (Table 1). The effect on mortality was also favourable: mortality due to severe cyanide poisoning decreased from 52.0% before the programme to 28.3% after. There were increases in the overall and appropriate use of antidotes for severe cyanide poisoning, both of which are independently associated with lower mortality.^11^

**Table 1:** Patients treated and annual budget of the Thai national antidote programme, 2011–2017

Year	Antidotes		Antivenoms		Total	
	No. of patients treated	Budget, US$^a^	No. of patients treated	Budget, US$^a^	No. of patients treated	Budget, US$^a^
2011	49	142 000	NA	ND	49	142 200
2012	106	422 000	NA	2 233 357^b^	106	422 000
2013	402	407 000	964	651 393	1366	1 058 393
2014	466	204 000	4966	1 675 677	5432	1 879 677
2015	191	252 000	6234	1 114 286	6425	1 366 286
2016	317	283 000	6824	1 140 286	7141	1 423 286
2017	269	223 000	6648	1 450 690	6917	1 673 690

NA: not applicable; ND: not determined; US$: United States dollar.

^a^ One United States dollar was equivalent to approximately 35 Thai baht between 2011 and 2017.

^b^ The average total annual cost of individual hospitals purchasing antivenoms during 2011 and 2012.

## Lesson learnt

The programme ensured timely access to essential antidotes and antivenoms, which saved lives even though the production and supply of these orphan drugs were limited and there were few clinical toxicologists in the country. Three factors contributed to the programme’s success (Box 2).

Box 2 **Summary of main lessons learnt**

- Strong and sustained policy support and full funding from the national budget ensured adequate supplies of essential antidotes and antivenoms.
- Improvements in operational capacity, which included central procurement, national and subnational antidote and antivenom stocks, and distribution aided by information and communication technologies, ensured these medicines were rapidly available for patients and minimized waste due to expired products.
- In-service training and 24-hour online consultations provided by poison centres improved clinical management and helped ensure antidotes and antivenoms were used correctly.

First, government policy was strong and sustained across different administrations, annual national budgets allocated full funding to the National Health Security Office for antidotes and antivenoms, which ensured an adequate supply of these essential medicines.

Second, the availability of antidotes and antivenoms was improved operationally by: (i) the use of central procurement; (ii) direct delivery from suppliers to national and subnational stocks; (iii) the creation of a management information system that included the number of doses available and their expiry dates; (iv) use of a web-based system to deal with requests from hospitals; and (v) timely delivery from stocks. These factors combined to ensure the timely use of antidotes and antivenoms and saved lives, even in very remote areas. In addition, the programme discouraged individual hospitals from purchasing and stocking these medicines, thereby decreasing wastage and substantially reducing costs.

Third, the manuals and clinical guidelines produced by poison centres and the annual training provided for clinicians improved case management and confidence in the use of antidotes and antivenoms. In addition, these centres provided 24-hour online consultation services to support case management remotely; therefore clinical toxicologists at the centres could observe patients’ clinical symptoms in real time.
